# Synergistic tribo-mechanical enhancement of heat-cured poly(methyl methacrylate) denture base via hybrid in-situ synthesized organic and inorganic nanoparticles

**DOI:** 10.1038/s41598-025-33026-2

**Published:** 2026-01-29

**Authors:** Howida Mohamed, W. Y. Ali, Abdallah Shokry, A. H. Badran, Ameer Ali Kamel

**Affiliations:** 1 Department of Mechanical Engineering, Faculty of Engineering, Fayoum University, Fayoum, 63514 Egypt; 2 Department of Production Engineering and Mechanical Design, Faculty of Engineering, Minia University, Minia, 61111 Egypt

**Keywords:** PMMA nanocomposites, Denture base material, Hybrid nanoparticles, Tribological performance, Mechanical properties, Engineering, Materials science, Nanoscience and technology

## Abstract

Poly(methyl methacrylate) (PMMA) is the primary material for dental applications, but it suffers from limitations such as poor wear resistance and long-term durability. To address these shortcomings, this work presents a new and cost-effective hybrid nanofiller system comprising hydroxyapatite (inorganic nanoparticles) and date seed (organic nanoparticles) for reinforcing PMMA denture bases. This study specifically investigates the reinforcement of heat-polymerized PMMA with this new hybrid nanofiller. Comprehensive material characterization was performed using XRD, DSC, SEM, TEM, and EDX. The findings demonstrate that PMMA composites with 0.2-1 wt% hybrid nanoparticles, particularly at 0.6 wt%, showed enhanced mechanical and tribological performance compared to the pure polymer. Specific improvements included a 30.99% decrease in the coefficient of friction, a 37.39% reduction in wear rate, a 31.92% increase in compressive strength, and a 9.87% improvement in surface hardness.

## Introduction

Partial or complete edentulism is a significant health issue that affects facial aesthetics, speech, chewing efficiency, dietary choices, and overall quality of life. Dental prostheses are commonly used to manage this condition, helping to restore both the visual appearance and functional capabilities of affected individuals^1^. Although, PMMA has been the predominant material for denture bases for over 70 years due to its excellent aesthetics, ease of fabrication, affordability, and biocompatibility^2^. Denture bases constructed from PMMA have several limitations. They are susceptible to breaking over time due to constant chewing pressure, and prolonged contact with saliva can worsen material wear, and possibly causing dental health issues. Furthermore, PMMA suffers from issues such as dimensional instability, limited mechanical strength, and polymerization shrinkage, which collectively reduce its long-term durability and clinical performance^3^. To address these limitations, recent advancements have concentrated on enhancing PMMA by incorporating nanofibers, functional fillers, and various chemical modifications^4,5^. These reinforcement strategies aim to improve the material’s toughness, mechanical strength, and overall reliability, thereby extending the service life and performance.

^6,7^. Furthermore, comprehensive reviews in the field consistently highlight the critical role of filler type, size, and concentration in determining the final performance of the composite material, underscoring the importance of systematic formulation^8–10^.

Building upon this foundation, recent investigations have shifted towards more complex, hybrid filler systems to achieve synergistic effects that are unattainable with single fillers. Studies have shown that hybrid composites, combining fillers of different sizes (micro-nano) or chemistries, can offer superior mechanical strength, enhanced wear resistance, and improved thermo-mechanical stability compared to their conventional counterparts^11–13^. The selection and development of dental materials have been advanced by Multi-Criteria Decision-Making (MCDM) methods. These techniques offer a structured approach to identifying optimal material formulations by systematically evaluating multiple competing properties^14–16^. Hybrid methods like ENTROPY-VIKOR^17,18^, FAHP-FTOPSIS^19^, and AHP-TOPSIS^20^ are particularly effective for this comprehensive ranking and selection process.

Hydroxyapatite (HA), with the chemical formula Ca₁₀(PO₄)₆(OH)₂, is a calcium phosphate compound and the main mineral component of bones and teeth^21^. Owing to its biocompatibility, non-toxicity, strong bioactivity, and osteoconductive nature, it is extensively employed in orthopedic applications such as bone regeneration and implants^22,23^. Additionally, HA is widely used as a dental restorative material and as a reinforcing filler in various medical composites, making it a pivotal biomaterial in the medical field^24^. Many studies have explored PMMA/HA composites, demonstrating notable improvements in fracture resistance and mechanical performance compared to pure PMMA. For instance, Wu et al.^25^ developed a reinforced PMMA composite incorporating nano-HA and nano-alumina, which exhibited enhanced fracture toughness. More significantly, Ataei-Aazam et al.^26^ conducted both numerical and experimental analyses on PMMA/HA nanocomposites, revealing improved mixed-mode (I/II) fracture resistance, thus addressing key limitations of pure PMMA.

Additionally, Kaloka et al.^27^ evaluated the efficacy of HA as a reinforcing filler in PMMA based denture materials. Their experimental study demonstrated that HA incorporation significantly enhances mechanical properties of denture bases, including hardness, flexural strength, and fracture resistance. Similarly, Aldabib and Ishak^28^ systematically examined how varying HA filler concentrations affect PMMA denture base characteristics. Their findings indicated that an optimal HA content significantly improves the composite’s flexural strength, modulus, and impact resistance, whereas excessive filler levels cause particle agglomeration, resulting in reduced mechanical performance. Yadav et al.^29^ demonstrated the difference between two dental composite types. The composite series incorporating alumina (Al₂O₃) with nHA consistently outperformed the one using titanium oxide (TiO₂) in terms of its thermo-mechanical and thermal degradation properties.

Exploring the potential of PMMA/HA composites in medical applications, the recent study by Saskianti et al.^30^ evaluated the antimicrobial properties of PMMA/HA composites against oral pathogens through in vitro testing. Their findings demonstrated the material’s significant antibacterial and antifungal activity, suggesting its potential for improving oral health applications in denture materials. Overall, these studies highlight HA’s effectiveness as a reinforcing additive in PMMA, delivering significant enhancements in mechanical strength, thermal stability, and antimicrobial functionality, making it highly suitable for dental and broader biomedical applications.

Eco-friendly composites reinforced with natural fibers and fillers have gained widespread use in numerous applications, driven by growing concerns over the environmental impact of synthetic materials. In recent decades, significant research has emphasized the development of sustainable and renewable resources that are biodegradable, non-toxic, cost-effective, and environmentally friendly. These natural reinforcements are sourced from various plant parts, offering diverse options for strengthening polymers and other materials^31^. Among these resources, the date palm stands out as an ancient crop that has been cultivated for thousands of years, with its origins traced back to Predynastic Egypt. Beyond its nutritional and cultural importance, it is increasingly being investigated for its byproducts (particularly date seeds) which show promise as versatile materials in engineering fields^32^. Recent research has specifically investigated the mechanical behavior of composites reinforced with date seeds, utilizing their distinctive properties to improve strength and durability.

For example, Adel et al.^33^ demonstrated that incorporating date seed microparticles into glass ionomer cement (GIC), the results showed that the addition of 5 wt% date seed (DS) reinforcement to GIC led to a marked enhancement in microhardness, impact strength, and compressive strength when compared with the unreinforced control GIC sample, especially under artificial saliva conditions. While, Nwogu et al.^34^ showed that date seed granulated powder (DSGP) can reinforce glass fiber reinforced epoxy (GFRE) composites, achieving optimal tensile and flexural strength at 50 wt% and 40 wt% DSGP, respectively. Meanwhile, Elkhouly et al.^35^ evaluated the mechanical properties of glass-epoxy composite systems reinforced with date seed (DS) fillers in comparison to two inorganic reinforcements silicon carbide (SiC) and aluminum oxide (Al₂O₃). Their findings showed that DS fillers exhibited competitive performance relative to the inorganic fillers while offering a more cost-effective alternative. Finally, for food packaging materials the innovative study by Lawal et al.^36^ developed sustainable, edible food packaging films using carboxymethylcellulose reinforced with date seed components. Their research demonstrated significant improvements in the films’ mechanical strength, physical characteristics, and bioactive properties, while maintaining edibility presenting a promising eco-friendly alternative to conventional.

Based on the reviewed literature, researchers have investigated the individual effects of HA NPs and DS NPs on the properties of PMMA. However, research on the synergistic influence of hybrid inorganic (HA) and organic (DS) nanofillers on the mechanical and tribological performance of PMMA remains limited. This study aims to harness the combined advantages of these hybrid fillers to enhance compressive strength, wear resistance, and hardness while preserving the material’s amorphous structure and biocompatibility. Also, this study aims to develop economical prepared organic nanostructures, such as DS NPs, as reinforcements for PMMA, with the additional goal of lowering the production costs of denture base composites by employing nano-organic fillers.

## Experimental procedures

### Base material

**Fig. 1:**
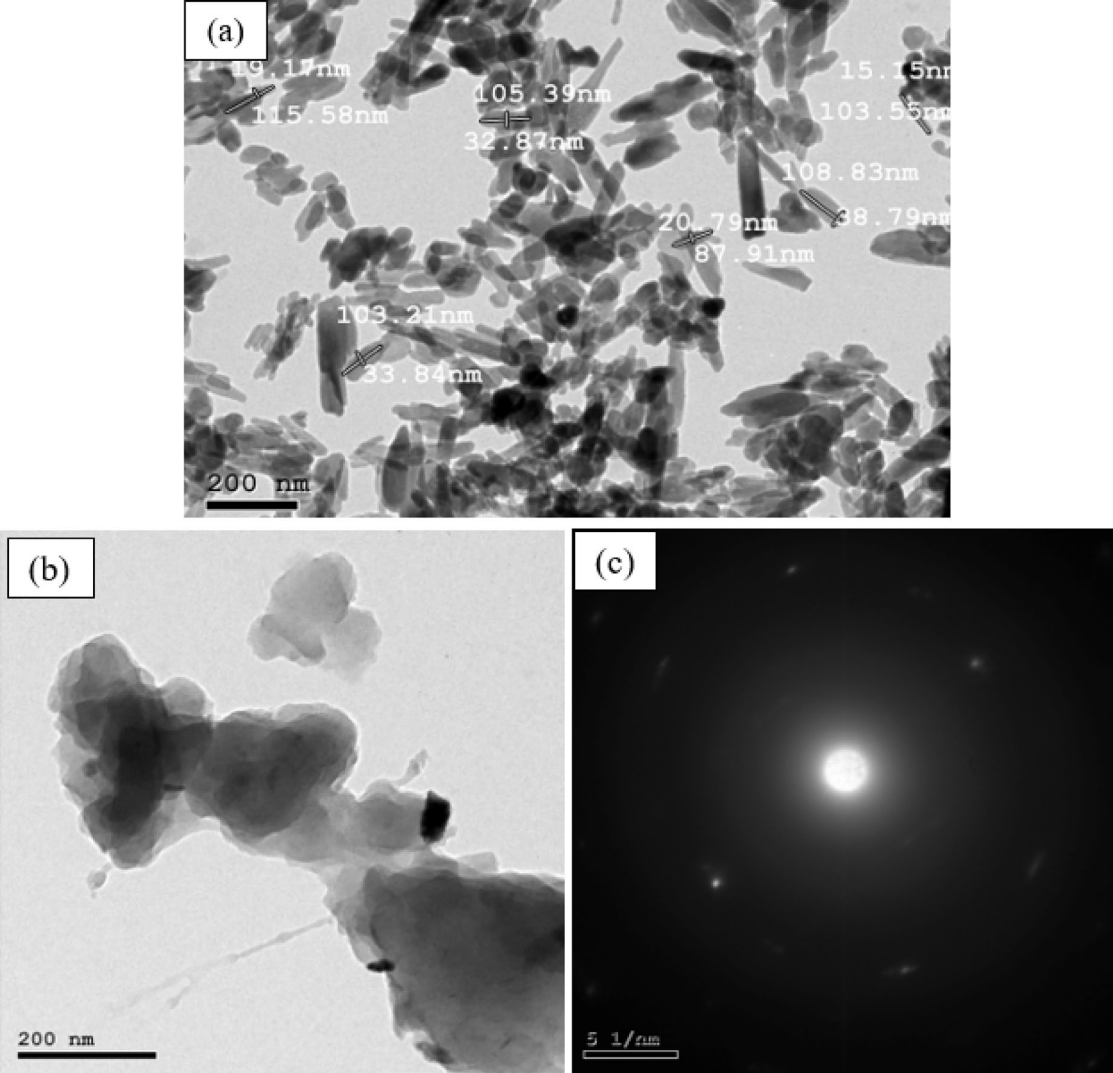
TEM image of (**a**) HA NPs (**b**) DS NPs and (**c**) TEM selected area electron diffraction (SAED) for DS NPs.

In this research, the denture base materials were made up of three key components: PMMA as a matrix material, HA NPs, and DS NPs as reinforcement materials. The PMMA material (heat cured) was supplied by Acrostone Dental & Medical Supplies Company, Cairo, Egypt, it has a density of 1.18 g/cm³. Methyl methacrylate (MMA), the liquid monomer used in PMMA synthesis, is a transparent and colorless substance with a characteristic density of 0.93 g/cm³ at standard conditions (25 °C, 1 atm). The HA NPs was supplied by Nano Tech company for Photoelectronics, Cairo, Egypt. The nanoparticles exhibit a rod-like shape with a white color and density of 3.02 g/cm³, measuring 100 ± 5 nm in length and 20 ± 3 nm in diameter as shown in Fig. 1a.

#### Preparation and structural characterization of the organic filler

The DS NPs were prepared by first washing the seeds thoroughly with fresh water, air-dried for 30 h, and then oven dried at 70 °C for 20 h to ensure complete dehydration. After drying, they were ground and ball-milled. Figure 1b presents TEM image of DS NPs, revealing a two-dimensional (2D) sheet-like or flake-like morphology. These nanostructures resemble graphene, nanosheets, or other layered materials and are often produced through mechanical processing techniques such as ball milling or ultrasonication, which break down bulk material into thin flakes^37^. Specifically, 2D materials have emerged as a central focus in materials science and chemistry, with extensive research devoted to their synthesis, characterization of properties, and exploration of technological and environmental applications^38^. Compared with spherical or bulk forms, 2D sheet-like nanoparticles exhibit exceptional advantages such as very high surface area, mechanical flexibility, and tunable properties. These features make them highly effective for applications in energy storage and harvesting, biomedical systems, and environmental remediation^39,40^. Additionally, TEM selected area electron diffraction (SAED) pattern (Fig. 1c) displays scattered bright spots and arcs arranged in concentric rings around the center, indicating diffraction from the crystalline planes of the nanoparticles. The appearance of these polycrystalline rings confirms that the Date seed nanoparticles exhibit well-defined crystallinity. The seeds were ground using a Ball milling process for 200 h, resulting in an average particle size of approximately 20 nm^41^.

### Sample Preparation

The methodological approach involved preparing a control sample of pure, heat-cured PMMA. This control was essential for comparative analysis against the reinforced PMMA nanocomposites that were fabricated next. The samples were made by mixing PMMA monomer (hardener liquid) with PMMA powder at a 1:2 ratio for 5 min. Hybrid nanofillers were then added to the mixture in concentrations of 0.2, 0.4, 0.6, 0.8, and 1 wt% (HA NPs to DS NPs ratio was kept at a 1:1 proportion). To ensure even particle distribution, the hybrid fillers were thoroughly mixed and stirred into the acrylic resin. The resulting nanocomposite blends were poured into cylindrical molds of 8 mm in diameter and 30 mm of height and compressed at room temperature under a pressure of 15 bar for 45 min. Then, the molds were subsequently placed in boiling water for 40 min, followed by gradual cooling to room temperature (bench cooling). The manufactured samples were trimmed to appropriate dimensions for further testing^42^. The nanocomposites, consisting of PMMA reinforced with varying amounts of HA NPs and DS NPs (0 to 1 wt%), were designated as HADS00 (0 wt%), HADS02 (0.2 wt%), HADS04 (0.4 wt%), HADS06 (0.6 wt%), HADS08 (0.8 wt%), and HADS10 (1wt.%).

### Tribological tests

**Fig. 2:**
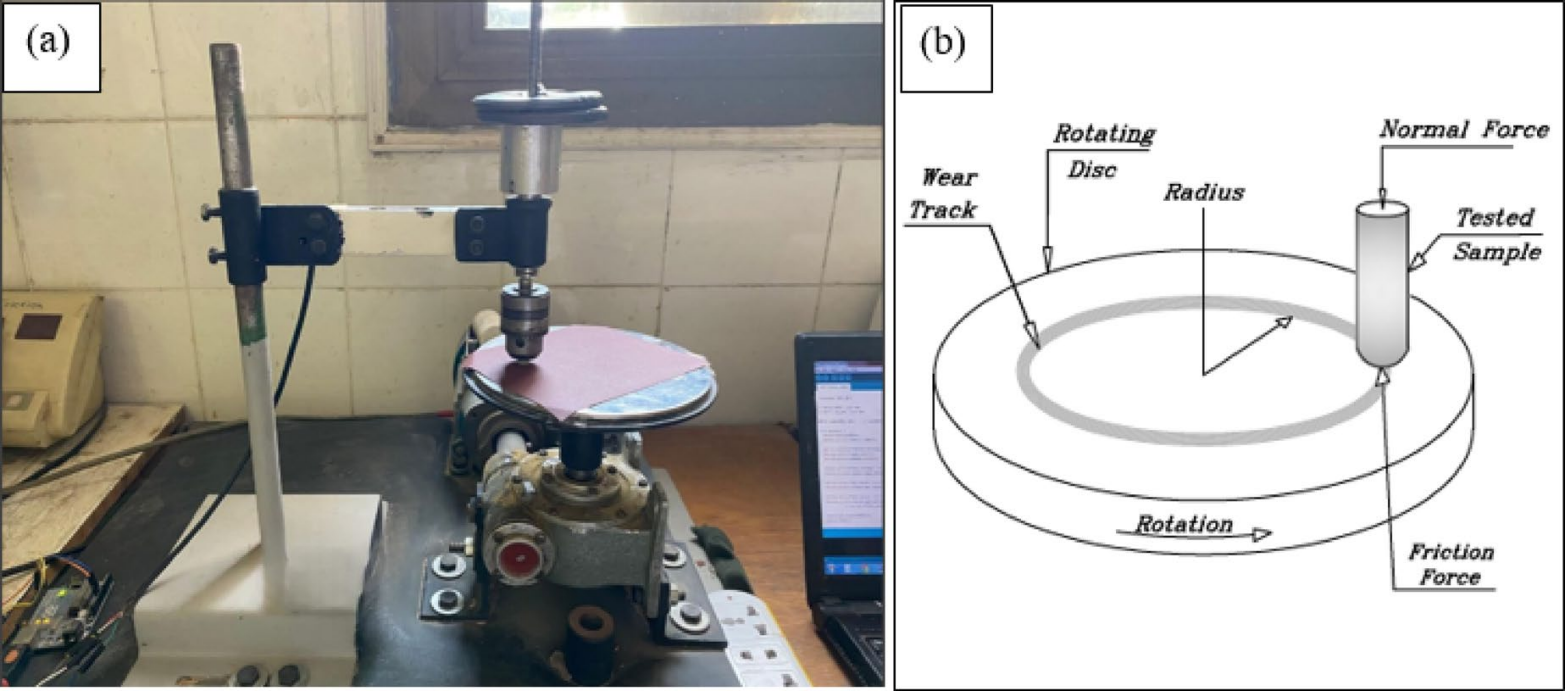
(**a**) Pin-on-disc tribometer, (**b**) Representation of the pin-disc tribological contact.

The wear behavior of the samples was evaluated based on ASTM G99-95, through gravimetric analysis using a high precision digital balance (± 0.0001 g accuracy), where mass loss was determined by measuring mass differences before and after testing. Friction and wear performance were evaluated using a pin-on-disc tribometer (made in Egypt) as shown in Fig. 2 with incremental normal loads (4–12 N in 2 N steps). The experimental configuration positioned the test material as a fixed pin in contact with a rotating disc with a sliding speed 0.4 m/s. Pin-on-disc testing was conducted against three characterized counterfaces:)1) 1000-grit SiC abrasive paper (adhesively fixed to the disc) featuring 14.5 μm silicon carbide particles with ~ 2500 HV hardness; 2) AISI 304 stainless steel (18–20% Cr, 8–10.5.5% Ni, balance Fe) with roughness (Ra = 0.025 μm, and Rz = 0.177 μm), 187 HV hardness, and 200 GPa Young’s modulus; and 3) cross-linked PMMA discs (fabricated from PMMA/benzoyl peroxide powder and MMA/EGDMA monomer) with roughness (Ra = 0.016 μm, and Rz = 0.151 μm), 165 HV hardness, and 2.8 GPa Young’s modulus. These distinct surface properties enabled comprehensive evaluation of the composite’s tribological behavior across different wear mechanisms.

### Mechanical tests

The mechanical properties were evaluated through hardness and compression tests. Surface hardness was measured using a Shore D durometer according to ASTM D2240. The instrument measures material resistance to indentation by a calibrated tip, with results reported on a dimensionless 0–100 scale. Five measurements were taken on each specimen surface, and the mean values were calculated^43^. Compressive strength was determined using a ZWICK Z010 universal testing machine following ASTM D695. Tests were conducted at a crosshead speed of 1.3 mm/min with a maximum applied load of 10 kN. This methodology provided quantitative assessment of the material’s resistance to compressive deformation.

### Thermal, structural, and microstructural analysis

X-ray diffraction analysis was carried out using a diffractometer (XRD Bruker D8 DISCOVER, Bruker AXS GmbH, Germany) with a step size of 0.0205° (2θ) over a scanning range of 3–70°. The thermal behavior of the samples was assessed by determining the glass transition temperature (Tg) through differential scanning calorimetry (Labsys DTA/DSC, Setaram, France), standard range: − 120 °C to 1600 °C. The DSC tests were performed within a temperature range of 0–350 °C, at a heating rate of 10 °C/min, using samples weighing approximately 17.71 mg. Furthermore, the surface morphology and wear features of the composite specimens were examined via scanning electron microscopy (QUANTA FEG 250, FEI, USA). This advanced high-resolution imaging method enabled precise assessment of two critical factors, the even distribution of nanoscale reinforcing particles throughout the polymer matrix, and the surface morphological changes induced by tribological wear testing. Transmission electron microscopy (JEOL JEM-2100, Japan) was employed to examine the morphology, dispersion, and crystallinity of HA NPs and DS NPs.

## Results and discussion

**Fig. 3:**
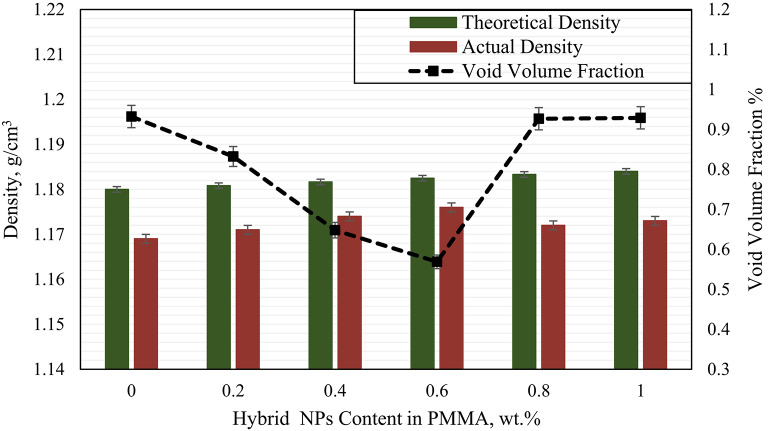
Theoretical and experimental densities of PMMA/hybrid NPs samples with different weight ratios.

The density of the composites was assessed using two complementary methods to ensure accurate characterization, theoretical estimation (ρ_th_, Eq. 1) and experimental determination via Archimedes’ principle^44^ (ρ_act_, Eq. 2). In the experimental method, the specimen’s mass was recorded both in air and when submerged in distilled water to calculate its actual density. The variation between ρ_th_ and ρ_act_, represented as the void volume fraction (Eq. 3), serves as a critical indicator of internal microstructure. A smaller difference indicates minimal porosity, uniform filler dispersion, and strong interfacial bonding. As shown in Fig. 3, the hybrid filler composite exhibits a maximum void volume fraction of 0.93%, which confirms a well consolidated material structure.

1 $$\:{\rho\:}_{th}=\frac{1}{\frac{{w}_{p}}{{\rho\:}_{p}}+\frac{{w}_{h}}{{\rho\:}_{h}}+\frac{{w}_{d}}{{\rho\:}_{d}}}$$

2 $$\:{\rho\:}_{act}=\left({\rho\:}_{water}+{\rho\:}_{air}\right)\times\:\frac{{m}_{S.air}}{{m}_{S.air}+{m}_{S.water}}+{\rho\:}_{air}$$

3 $$\:{V}_{v}=\frac{{\rho\:}_{th}-{\rho\:}_{act}}{{\rho\:}_{th}}$$

Where *w*_*p*_,*w*_*h*_ and *w*_*d*_ are loading amount of PMMA resin, HA NPs and DS NPs, respectively. *ρ*_*P*_, *ρ*_*h*_ and *ρ*_*d*_ are the densities of components. *ρ*_*water*_ and *ρ*_*air*_ are the densities of water and air, respectively. *m*_*s*.*air*_ and *m*_*s*.*water*_ are masses of the samples on each media.

### Tribological performance

**Fig. 4:**
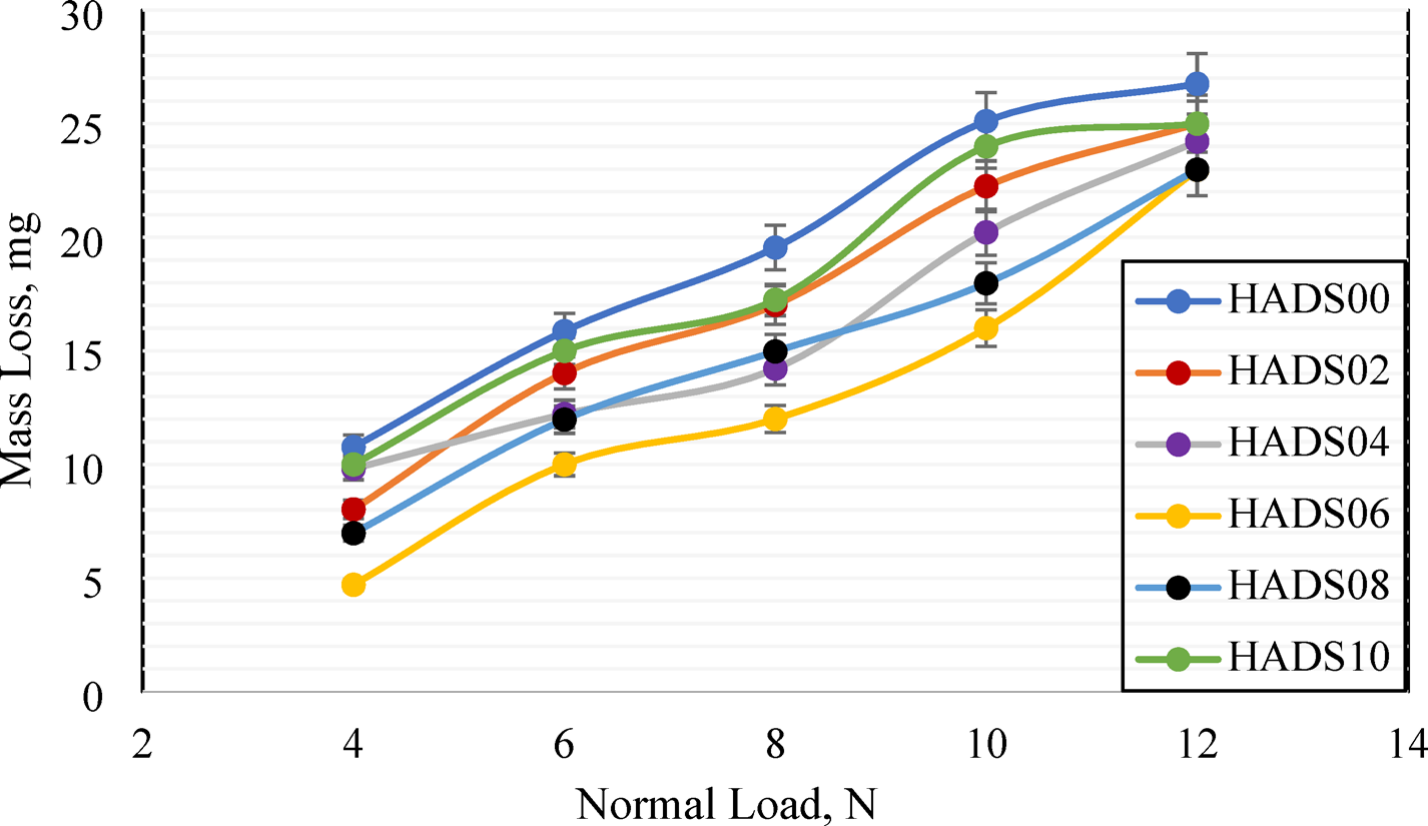
Mass loss of PMMA/hybrid NPs samples with different weight ratios and loads sliding against emery paper counterface.

The samples were tested against emery paper (1000 grit size) to evaluate wear behavior, with mass loss measurements recorded under varying normal loads of 4, 6, 8, 10, and 12 N. Figure 4 illustrates the relationship between normal load (N) and mass loss (mg). Across all loading conditions, pure PMMA composite exhibited the highest mass loss. As the concentration of hybrid nanoparticles increased, mass loss progressively decreased, reaching a minimum at HADS06, particularly under 4 N load indicating optimal tribological performance. This enhancement is due to the even distribution of nanoparticles in the PMMA matrix, which results in a smoother surface and reduces abrasive wear^45^.

Increasing the hybrid nanoparticle concentration to 0.8–1.8 wt% led to higher mass loss, especially under higher loads. At these filler levels, nanoparticle agglomeration took place. When these clustered particles interacted with the abrasion disc, they were gradually removed, initiating three-body abrasion and further accelerating wear^46^. Also, as illustrated in Fig. 4, the mass loss rises with the increase in the applied load, this occurs because greater normal loads enlarge the actual contact area between surfaces, enhancing adhesion and promoting material transfer. Additionally, higher loads produce more frictional heat, which softens the polymer matrix and increases its susceptibility to wear^47^.

From wear results, reinforcing the PMMA composite with 0.6 wt% of hybrid nanoparticles significantly enhances its tribological performance. For instance, several studies support these results, suggesting that the best performance is achieved at filler loadings of 0.4–1 wt.%^48,49^.

**Fig. 5:**
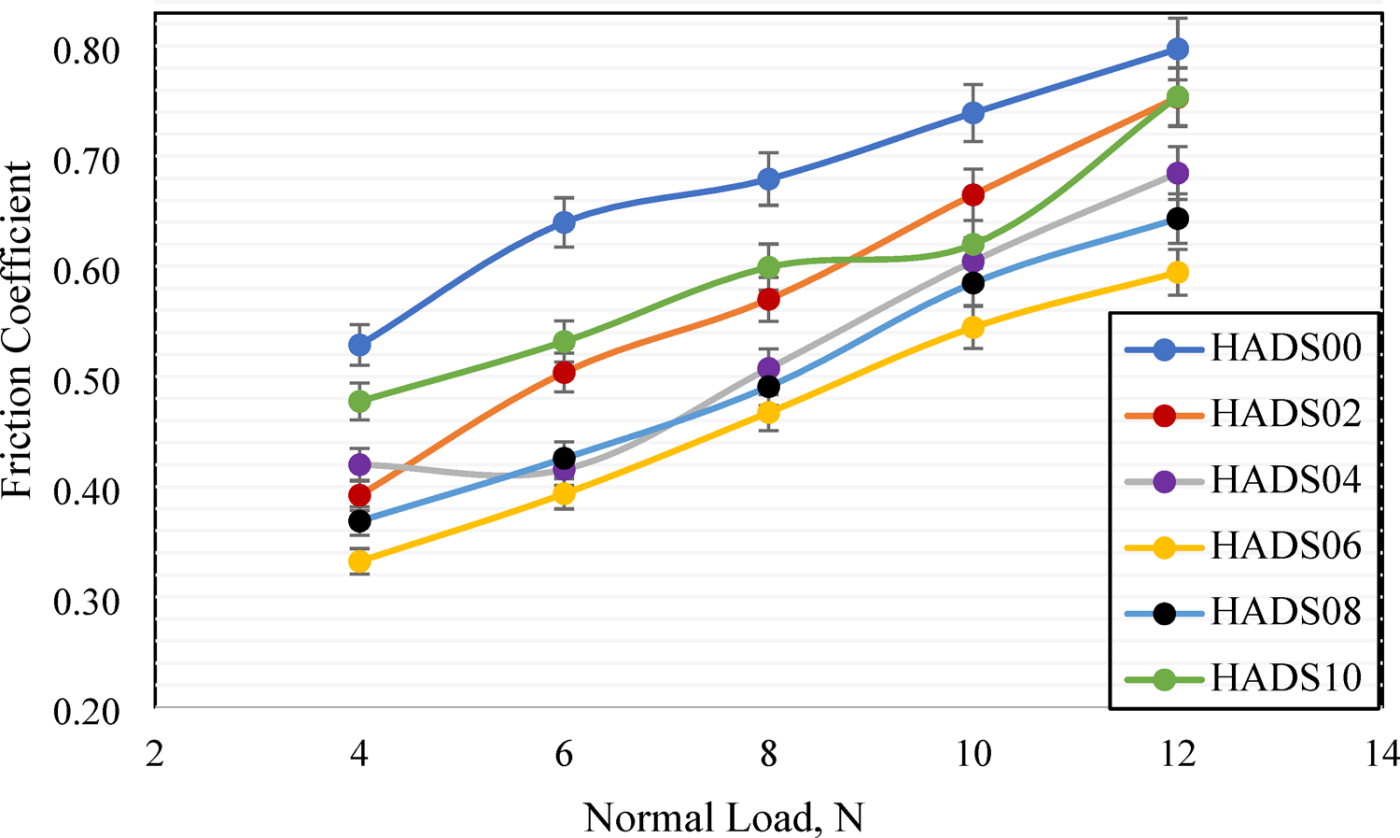
Friction coefficient of PMMA/hybrid NPs samples with different weight ratios and loads sliding against emery paper counterface.

Figure 5 demonstrates the impact of hybrid nanoparticle concentration on the frictional behavior of PMMA naoncomposites. The experimental results for COF quantitatively represent the wear behavior, showing a reduction in COF for all nanoparticle reinforced samples relative to pure PMMA, with the best performance observed at HADS06. The composite materials exhibited COF reductions of 22.12% (HADS04), 30.99% (HADS06), and 25.63% (HADS08), demonstrating the effectiveness of nanoparticle reinforcement in enhancing tribological properties.

The observed friction reduction is facilitated by the ability of the nanoscale hybrid particles to access the asperities of the sliding interface. By occupying these contact points, the particles reduce the real area of contact, inhibit adhesive junction formation, and facilitate inter-particle shear at the interface-a process that demands less energy than the bulk polymer deformation, thus providing an effective self-lubricating effect^50^. The analysis indicates that the COF increases with higher applied loads as a result of thermally induced surface softening. This softening expands the real contact area and enhances interfacial adhesion under elevated normal forces, thereby causing higher friction levels^51^. In addition, the applied normal loads of 4, 6, 8, 10, and 12 N corresponded to contact pressures of approximately 79.6 kPa, 119.4 kPa, 159.2 kPa, 198.9 kPa, and 238.7 kPa, respectively, based on the nominal contact area of 50.27 mm² for the 8 mm diameter pin.

**Fig. 6:**
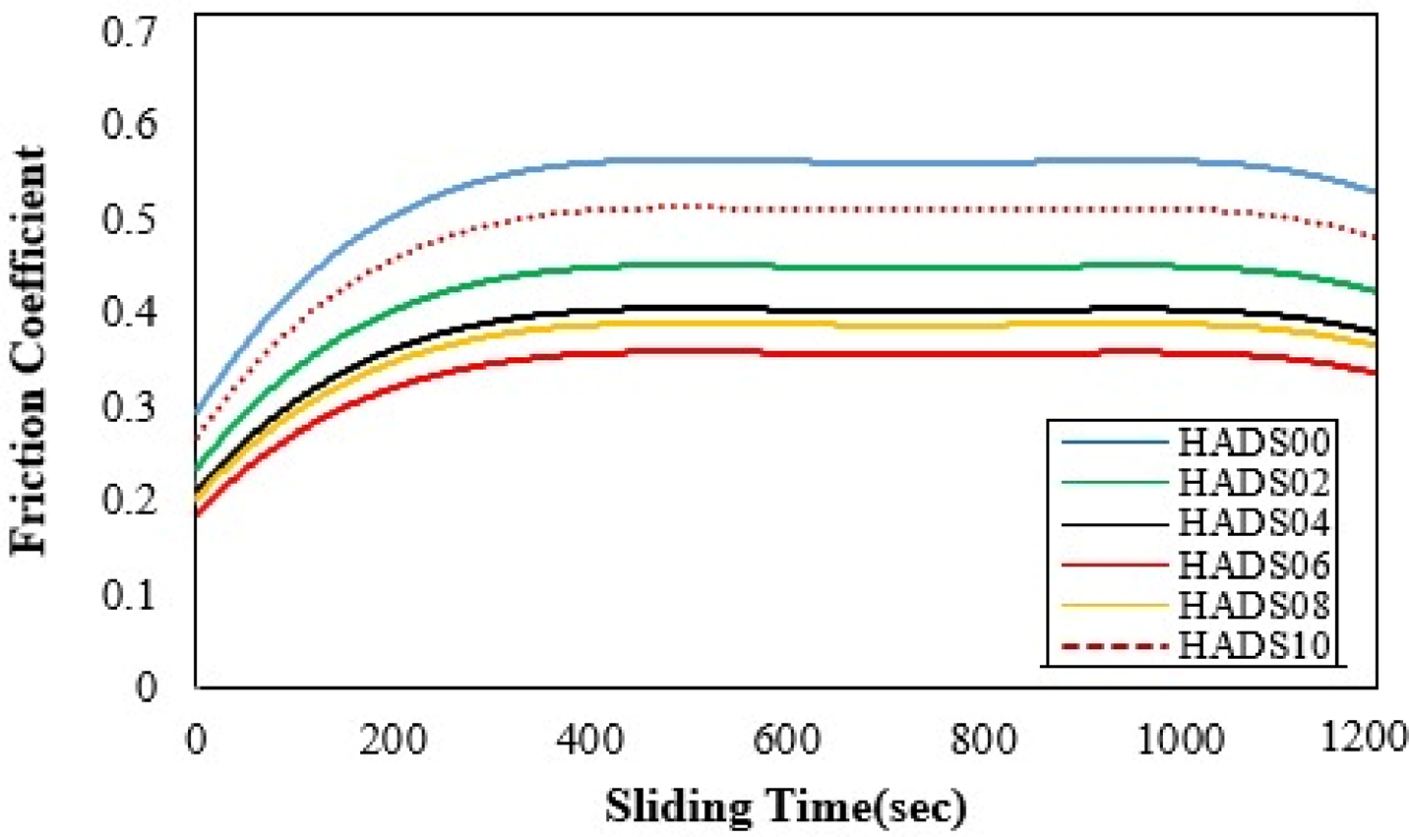
COF vs. time of PMMA/hybrid NPs samples with different weight ratios.

The COF vs. time plots further clarify the wear behavior (see Fig. 6). They show either (a) a gradual decrease in COF, which is consistent with adhesive transfer-film formation, or (b) an increase in COF when agglomerated particles are present, which is consistent with three-body abrasion. Therefore, the three-body abrasive mechanism is not invoked as a general mechanism for all loads and compositions; rather, it is specific to conditions involving high filler content, higher loads, and abrasive counter faces where clustered particles detach and form a rough third body.

**Fig. 7:**
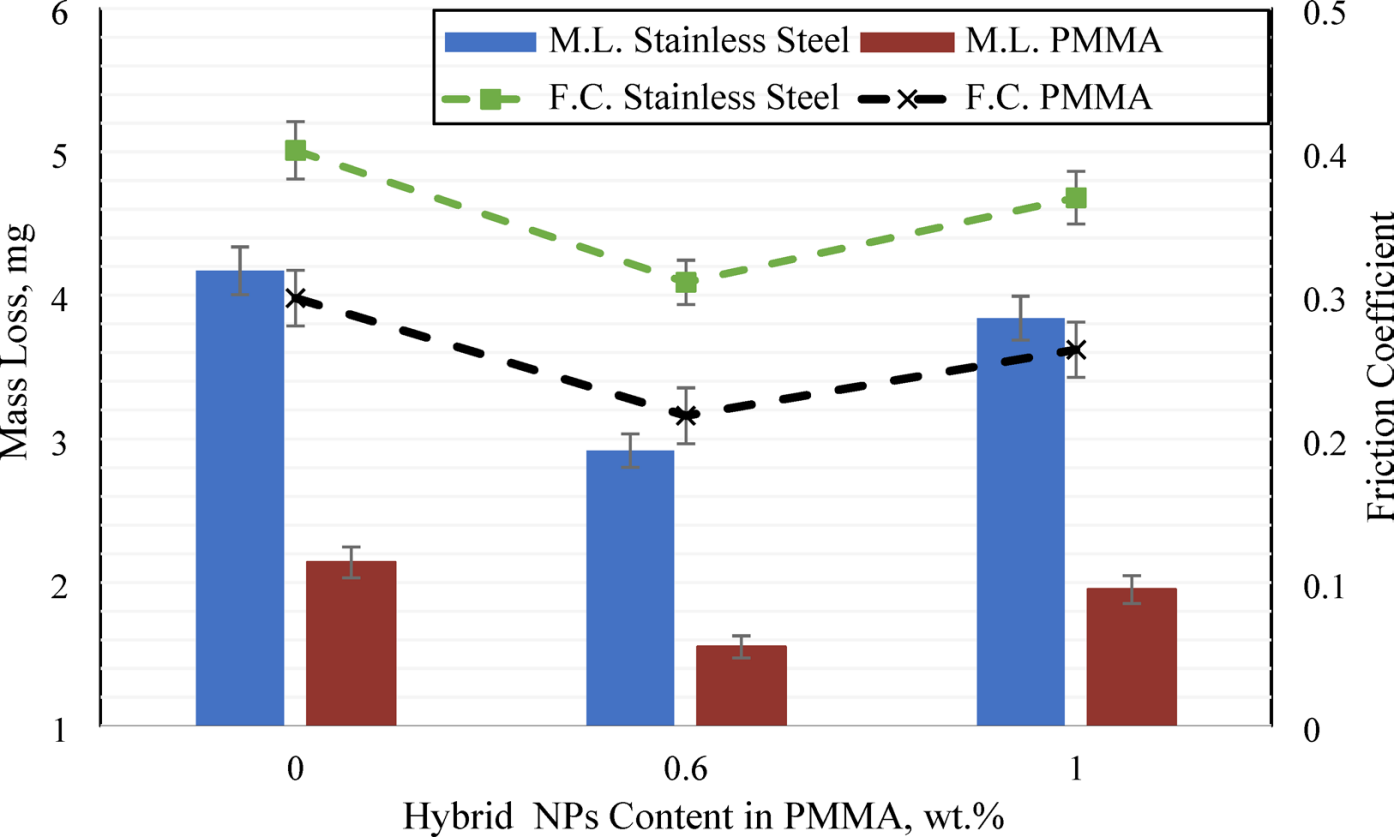
Mass loss and friction coefficient of HADS00, HADS06, and HADS10 sliding against stainless steel and PMMA counterfaces.

To comprehensively evaluate the wear performance and underlying mechanisms, the influence of counterface material was systematically investigated, as summarized in Fig. 7. Employing multiple counterfaces enabled the assessment of distinct wear modes including abrasive and adhesive mechanisms and confirmed the efficacy of nanoparticle reinforcement across varied tribological conditions. This methodological approach yields a robust, and clinically relevant understanding of the composite behavior. The mass loss of unreinforced PMMA, along with the highest and lowest mass loss values recorded for all nanocomposites when tested against emery paper, was further evaluated against stainless steel and PMMA counterfaces. As shown in Fig. 7, the results demonstrated consistent tribological behavior across counterfaces materials, with the improvements observed against stainless steel and PMMA closely reflecting those previously noted for emery paper. Wear and coefficient of friction were characterized for compositions HADS00, HADS06, and HADS10 across a load range of 4 to 12 N, with reported values representing the mean of the measurements. Against stainless steel, mass losses were 4.17 mg (HADS00), 2.92 mg (HADS06), and 3.84 mg (HADS10). When tested against PMMA, average weight losses decreased to 2.14, 1.55, and 1.95 mg, respectively. Sample HADS06 exhibited the most notable improvement, with COF reductions of 22.94% against stainless steel and 27.51% against PMMA.

**Fig. 8:**
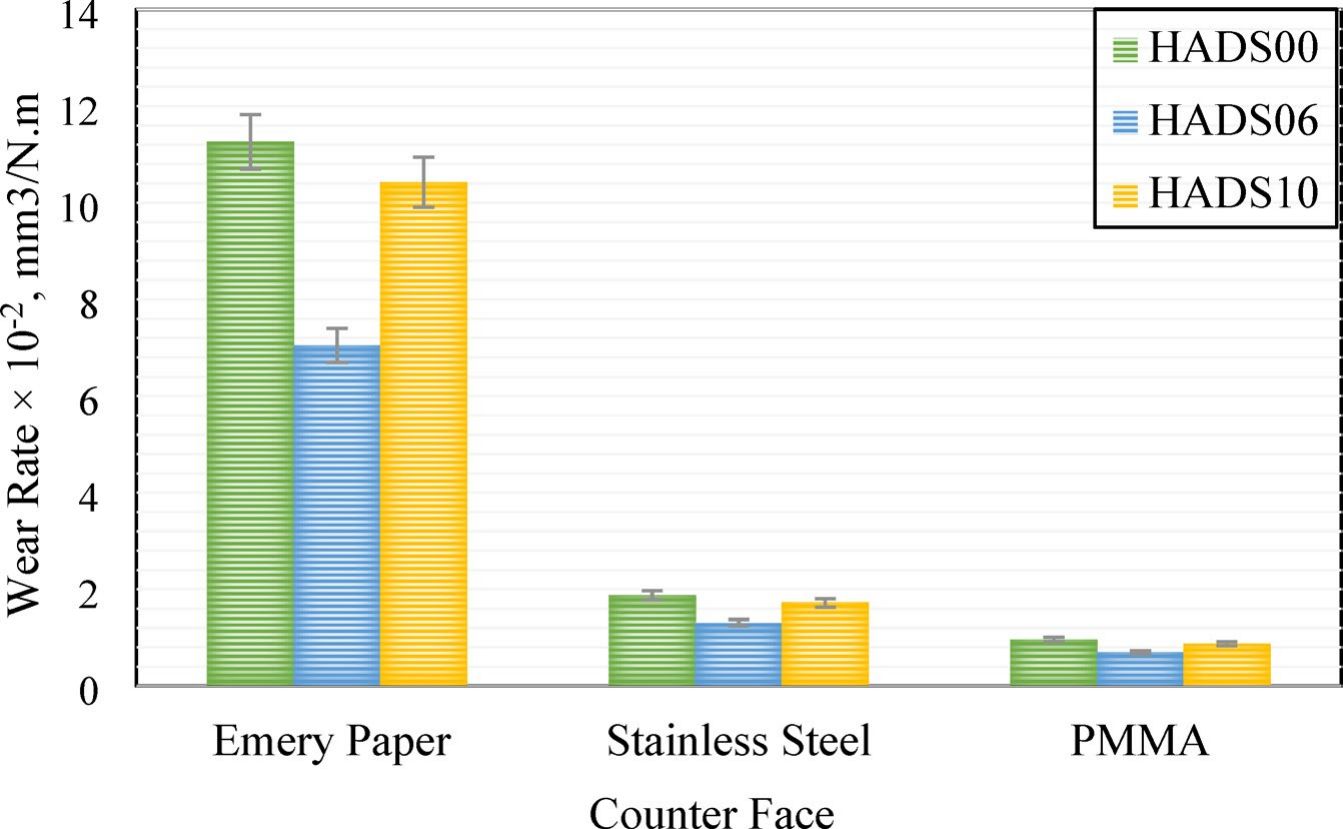
The effect of counter face on the wear rate of samples HADS00, HADS06, and HADS10.

The wear rates of composites HADS00, HADS06, and HADS10, calculated using Eqs. 4, 5, are presented in Fig. 8 with respect to counter face type. The results demonstrate that wear severity is highly dependent on the counterface material, with emery paper producing the most aggressive wear, followed by stainless steel, and finally PMMA. Across all counterface materials, the unmodified composite (HADS00) consistently displayed the greatest wear rate. Conversely, the nanocomposite with 0.6 wt% hybrid nanoparticles demonstrated a marked decrease in wear rate, achieving reductions of 37.39% on emery paper, 30.15% on stainless steel, and 27.75% on the PMMA counterface when compared with the control.

4 $${W_r} = \;\frac{{\Delta m}}{{d.\rho .{F_n}}}$$

5 $$d = 2\pi r \cdot \omega \cdot t$$

Where $\:\varDelta\:m$ is the mass loss, d represents the sliding distance, ρ is the material density, and Fₙ is the applied normal load. The parameters include *r* for the wear track radius (2.5 cm), *ω* for the rotational speed (2 rev/sec), and *t* for the test duration (60 s).

**Fig. 9:**
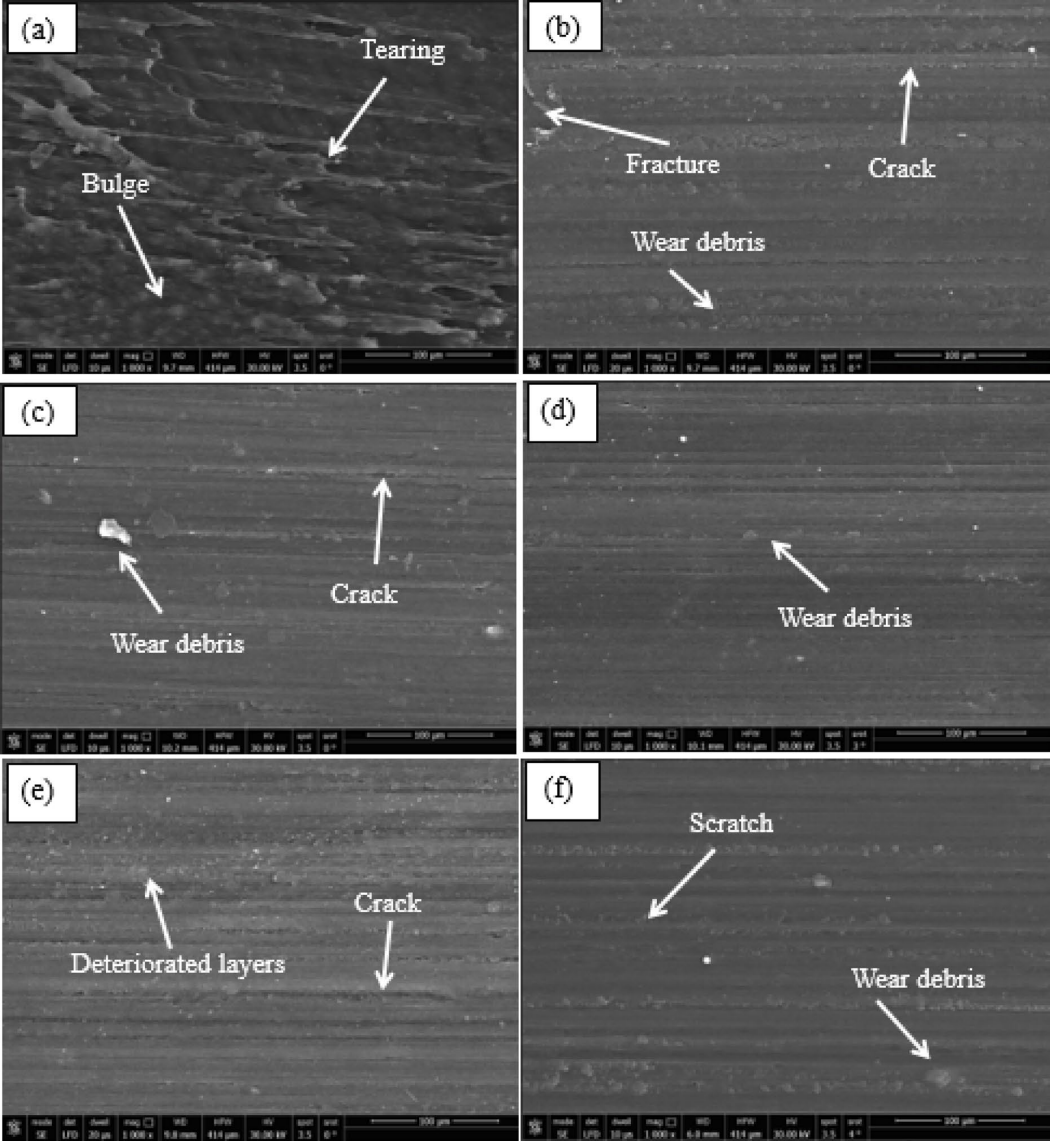
SEM micrographs of the worn surfaces for (**a**) HADS00, (**b**) HADS02, (**c**) HADS04, (**d**) HADS06, (**e**) HADS08 and (**f**) HADS10.

Scanning electron microscopy (SEM) was employed to examine the worn surfaces of both unmodified and hybrid nanoparticle reinforced PMMA composites in order to identify the prevailing wear mechanisms. As shown in Fig. 9a, pure PMMA exhibits an irregular worn surface morphology characterized by numerous tearing flakes and pronounced surface bulges. This observation aligns with earlier findings of increasing wear and friction behavior in HADS00 sample. The findings further demonstrate that unmodified PMMA predominantly undergoes adhesive wear on its worn surface, this conclusion is supported by the findings in^52,53^. However, increasing the hybrid filler content to 0.6 wt% led to better stress transfer between the polymer matrix and the nanoparticles, which in turn produced smoother wear tracks and shallower scratches (Fig. 9d). At samples HADS04 and HADS08 (Figs. 9c, e), close inspection reveals the presence of shallow scratching patterns and wear debris across the worn surface. Furthermore, they increase at samples HADS02 and HADS10 (Figs. 9b, f).

Tribological testing against three counterfaces (emery paper, stainless steel, and PMMA) under 4–12 N normal loads revealed a strong correlation between counterface material and the dominant wear mechanism. While abrasive wear with distinct plowing grooves characterized contact with emery paper, the stainless-steel disc prompted a mixed adhesive-abrasive mode. Conversely, PMMA contact led predominantly to adhesive wear. These results underscore the efficacy of the hybrid nanoparticles in strengthening the composite, as evidenced by improved interfacial adhesion, enhanced load distribution, and reduced wear across all test conditions. This establishes a direct link between the nanocomposite’s formulation and its performance under diverse tribological scenarios.

### Mechanical properties

**Fig. 10:**
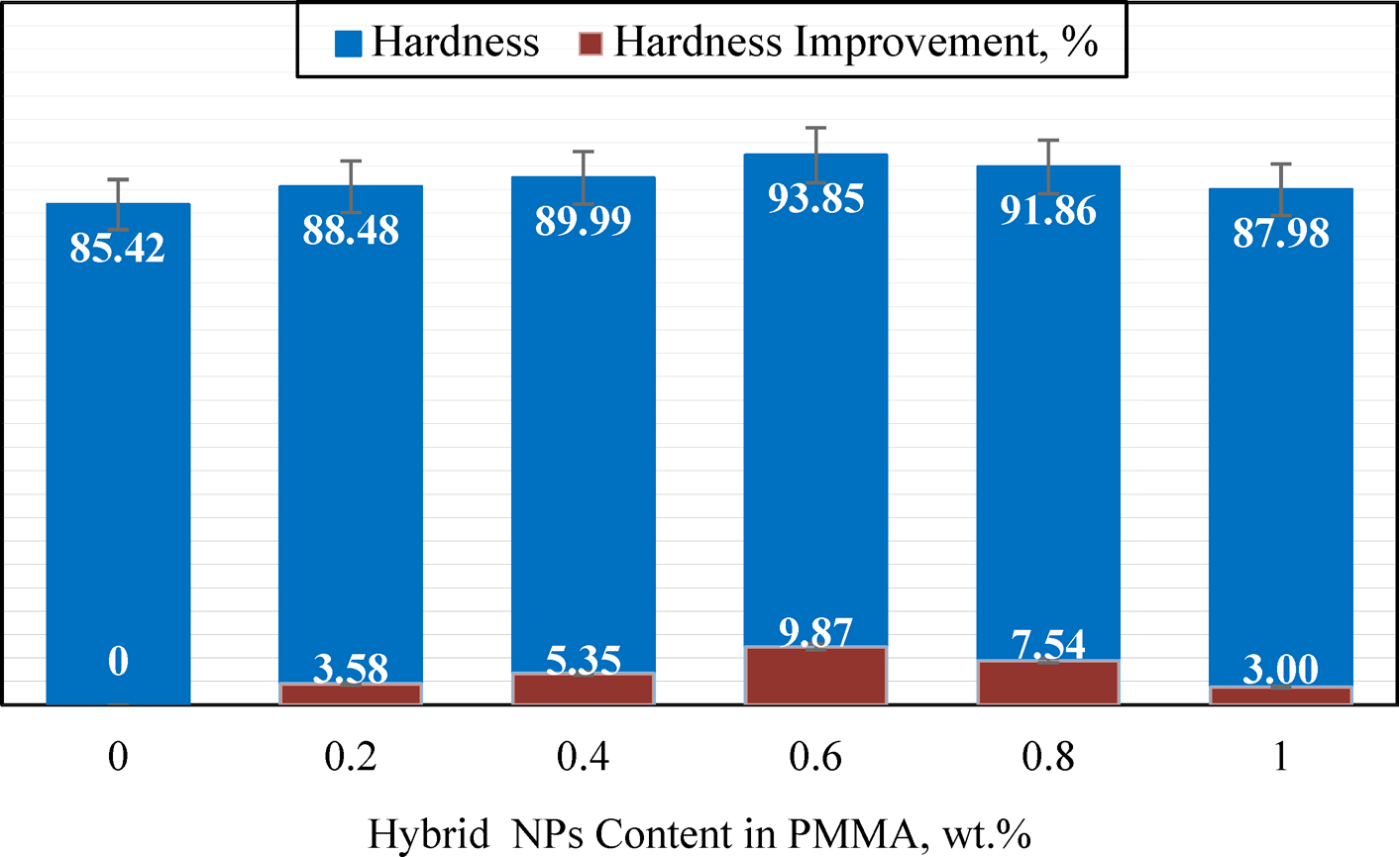
Hardness of PMMA/hybrid NPs samples with different weight ratios.

Denture bases experience continuous mechanical stresses during normal functions like mastication, oral hygiene maintenance, and regular use. Enhanced surface hardness in denture base materials provides greater resistance to surface damage from abrasion, scratches, and wear, thereby improving longevity and clinical performance. Figure 10 demonstrates the hardness improvement achieved through hybrid nanoparticle reinforcement. The pure PMMA recorded a baseline hardness of 85.42 (D index), while the incorporation of 0.6 wt% nanoparticle loading exhibited a 9.87% increase to 93.85 (D index). Furthermore, the composite’s hardness deterioration at high filler content stemmed from particle clustering via van der Waals forces, which weakened interfacial bonding effectiveness^54^.

**Fig. 11:**
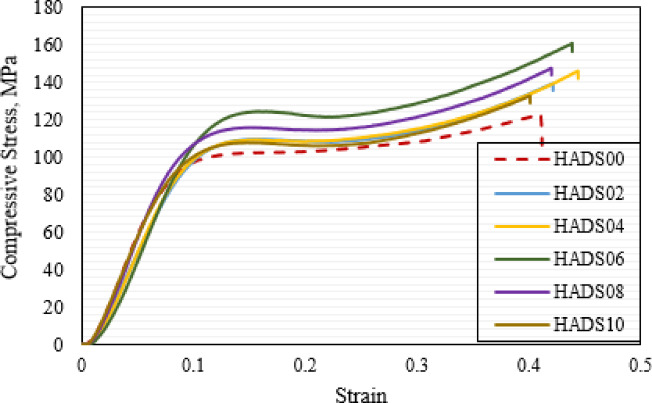
Stress–strain curves of PMMA/hybrid NPs samples with different weight ratios.

**Fig. 12:**
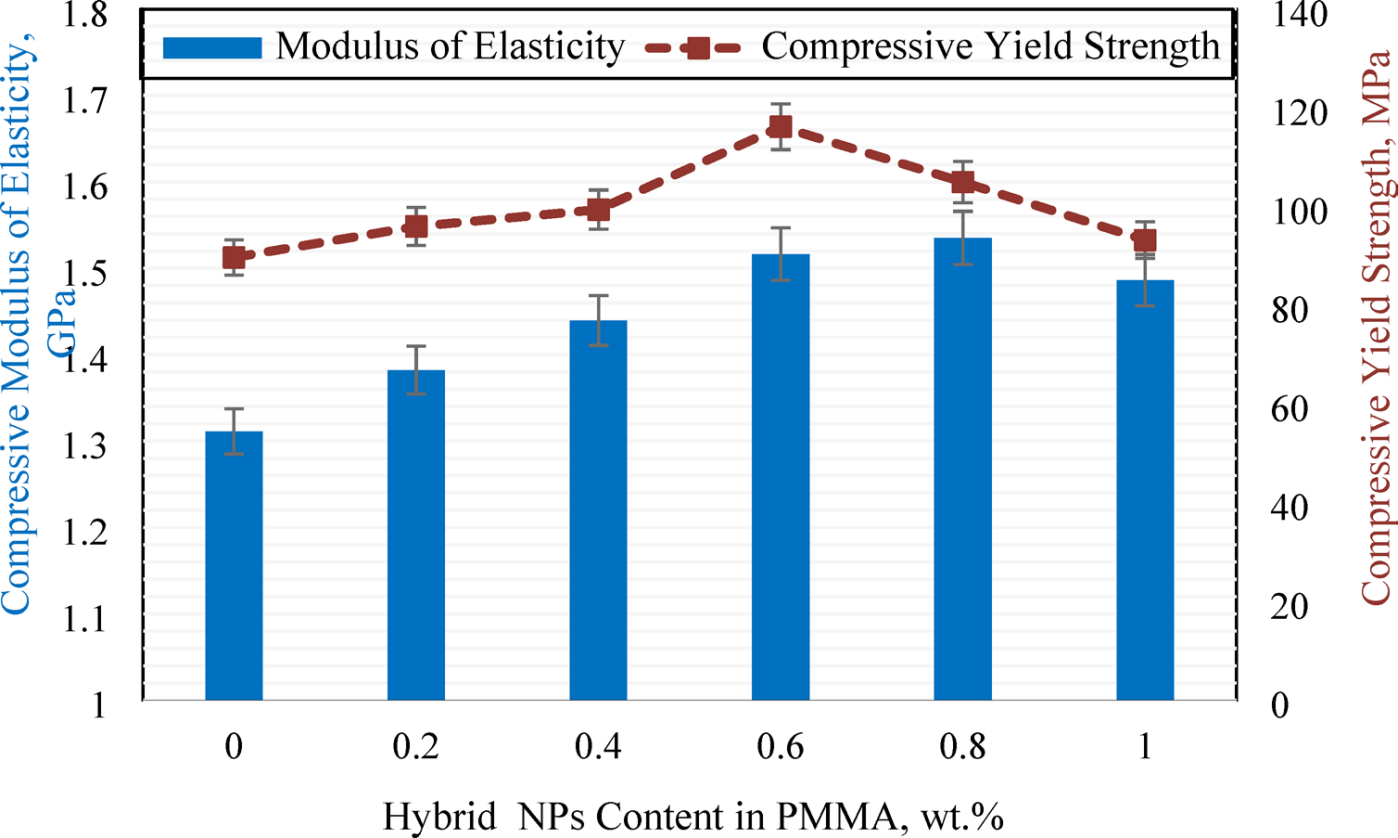
Modulus of elasticity and compressive yield strength of PMMA/hybrid NPs samples with different weight ratios.

The stress-strain curves presented in Fig. 11 demonstrate the material’s behavior under compressive loading and its deformation characteristics, while highlighting the influence of hybrid nanoparticle incorporation on the mechanical performance of PMMA denture base material. As illustrated in the figure HADS00 exhibited a compressive strength of 122.13 MPa. The incorporation of hybrid nanoparticles significantly enhanced this property, with the maximum compressive strength of 161.12 MPa achieved at sample HADS06 with a 31.92% improvement over pure PMMA. As shown in Fig. 12, this sample also yielded the highest compressive yield strength of 116.15 MPa (1.52 GPa elastic modulus), representing a 29.51% increase from the base material’s yield strength of 89.68 MPa (1.31 GPa elastic modulus).

The performance peak at the 0.6 wt% filler concentration reveals a synergistic reinforcement mechanism from co-incorporating DS and HA NPs, surpassing the effectiveness of single fillers. This conclusion is substantiated by comparative literature: Salih et al.^55^ reported maximum compressive strengths of 120 MPa for PMMA/3 vol% HA and 140 MPa for PMMA/3 vol% TiO₂, while Elkhouly et al.^41^ reported 145 MPa and 120 MPa using 1.2 wt% DS and 1.2 wt% TiO₂, respectively. Our hybrid system achieves a strength of 161 MPa.

The improved properties of HADS06 result from an optimal nanoparticle concentration that maximizes reinforcement while preserving the matrix. The uniformly dispersed fillers strengthen the composite through two primary mechanisms: strong interfacial bonding for effective stress transfer and resistance to deformation, and the restriction of polymer chain mobility, which enhances stiffness and hardness.

**Fig. 13:**
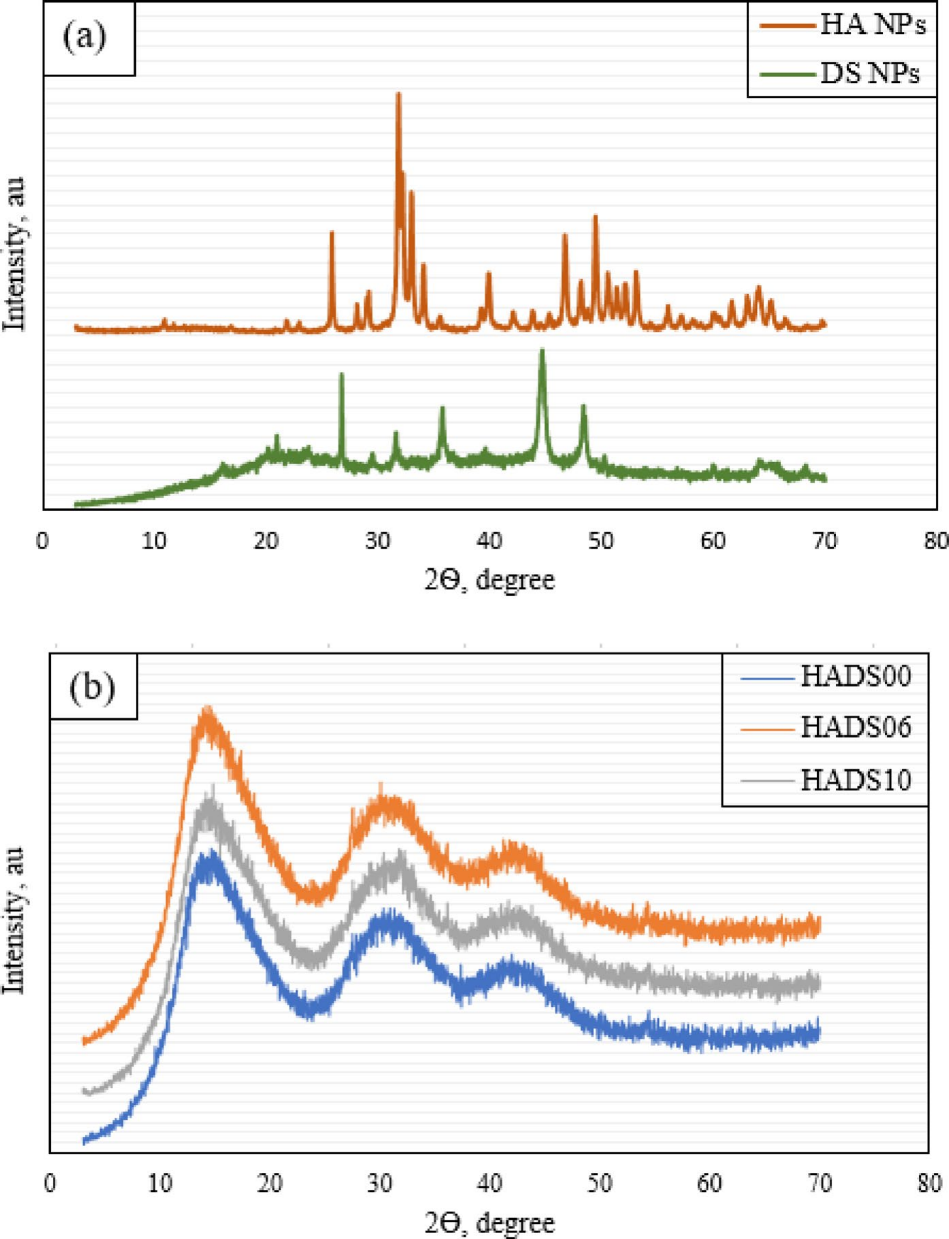
X-ray diffraction patterns (**a**) HA NPs, and DS NPs, (**b**) Samples HADS00, HADS06, and HADS10.

X-ray diffraction (XRD) stands as a fundamental characterization technique in materials science, provides critical structural information through diffraction pattern analysis^56^. The XRD patterns of HA NPs and DS NPs are presented in Fig. 13a. The sharp diffraction peaks of HA NPs indicate their high crystallinity and phase purity, whereas the broader peaks of DS NPs suggest a semi-crystalline, carbon-rich structure originating from their biogenic source. Figure 13b displays the XRD spectra of pure PMMA and its nanocomposite variants (HADS06 and HADS10). In agreement with previous findings, pure PMMA shows a typical amorphous pattern characterized by three broad peaks one major peak at 14.5° and two minor ones at approximately 30.9° and 41.3°^57^. Such broad diffraction features are characteristic of PMMA’s amorphous structure, with the lack of sharp peaks further confirming its non-crystalline nature^58^. The XRD patterns of the composites were consistent with pure PMMA, confirming the preservation of its amorphous structure. The absence of new crystalline peaks indicates that the hybrid nanoparticles were physically dispersed within the polymer matrix without forming chemical bonds or altering its fundamental architecture, which aligns with established literature.

**Fig. 14:**
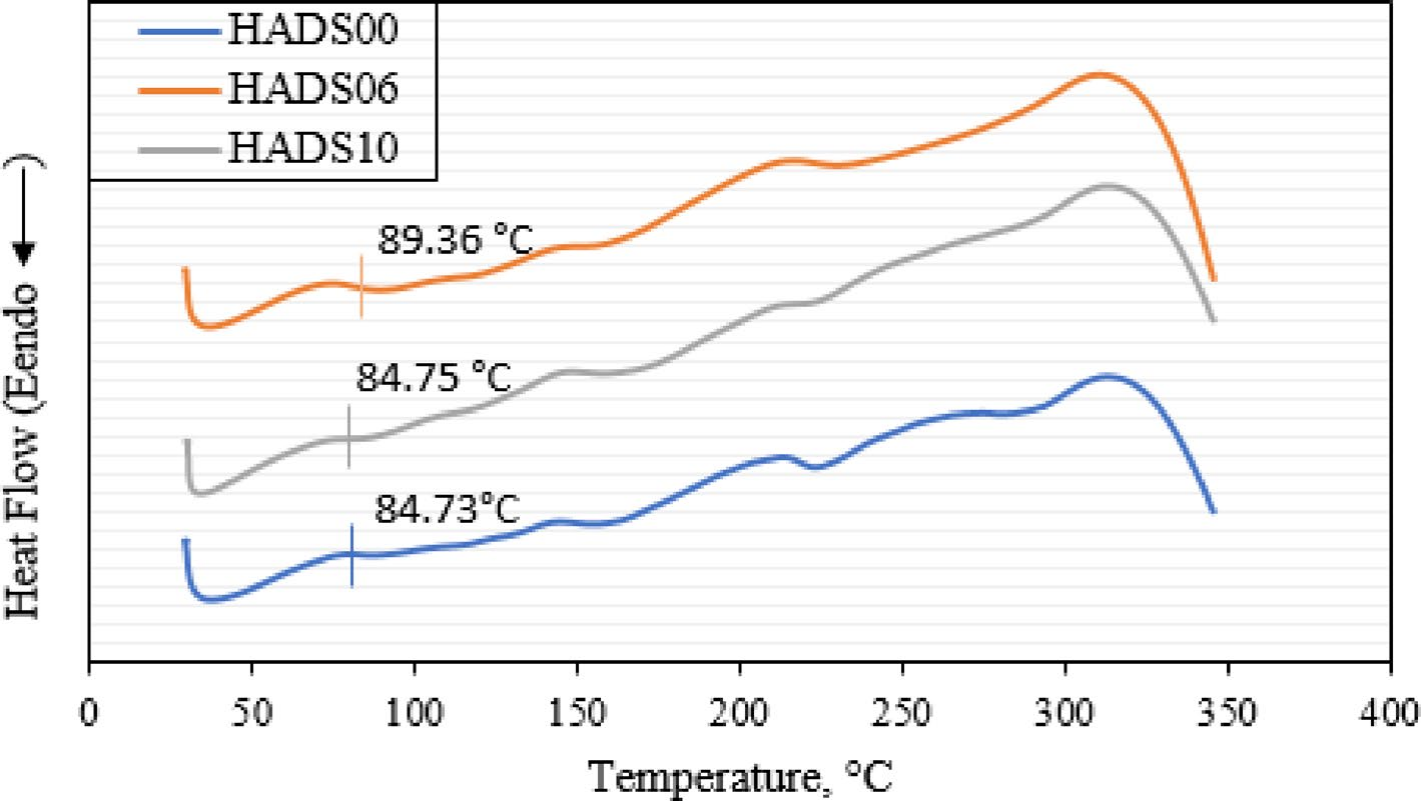
Differential scanning calorimetry curves of samples HADS00, HADS06, and HADS10.

When a polymer is incorporated into a substrate, its properties may be influenced by molecular interactions with the reinforcing nanoparticles. These interactions can modify the polymer’s glass transition temperature (Tg)^60^. The actual DSC measurements were conducted within the range of 0–350 °C. This range is appropriate for evaluating Tg and thermal behavior of PMMA-based composites, remaining well below the material’s decomposition temperature. As shown in Fig. 14, the thermal behavior was characterized using differential scanning calorimetry (DSC) for the neat polymer and composites with 0.6 wt% and 1.0 wt% filler concentrations. In contrast, the Tg of the HADS10 composite (84.74 °C) was similar to that of the neat PMMA control (HADS00), whereas HADS06 exhibited a higher Tg (89.36 °C) as shown in Fig. 14. This increase suggests that at 6 wt%, the hybrid nanoparticles strengthen interfacial interactions with PMMA chains, restricting their mobility and raising the Tg relative to pure PMMA.

**Fig. 15:**
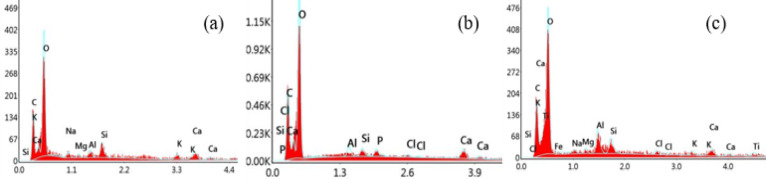
Energy dispersive X-ray spectroscopy of samples (**a**) HADS00, (**b**) HADS06, and (**c**) HADS10.

The chemical composition of the nanocomposite was determined using energy dispersive X-ray spectroscopy (EDX), with the results presented in Fig. 15. The EDX spectrum for pure PMMA (Fig. 15a) confirms the presence of carbon (C) and oxygen (O) in its structure. This is consistent with the molecular composition of PMMA, as hydrogen (H) is undetectable by standard EDX systems. The spectra for the PMHD06 and PMHD10 composites (Fig. 15b and c, respectively) reveal the presence of calcium (Ca) and phosphorus (P). in addition to the carbon and oxygen signals which remain stronger than in the pure PMMA spectrum (Fig. 15a) confirming the successful incorporation of both hydroxyapatite and date seed nanoparticles.

## Conclusion

This study investigated an approach to mitigate the inherent limitations of poly(methyl methacrylate) for denture bases by incorporating a hybrid nanofiller of hydroxyapatite and date seed nanoparticles at loadings of 0.2–1 wt%. Comprehensive material characterization using XRD, DSC, SEM, and TEM confirmed the successful integration and structural integrity of the composite.

Among the tested compositions, the formulation with 0.6 wt% hybrid nanoparticles (HADS06) demonstrated the most enhancement, which exhibited a 31.92% rise in compressive strength and a 9.87% increase in Shore D hardness, suggesting enhanced resistance to deformation. Tribological properties were also achieved a 37.39% reduction in wear rate and a 30.99% lower coefficient of friction compared to unreinforced PMMA. Furthermore, the nanocomposite exhibited enhanced wear resistance not only under harsh abrasive conditions (with emery paper) but also in smooth metallic interactions (with stainless steel) and in self-mated sliding (with PMMA), highlighting its durability and suitability for a wide range of dental applications.

This study demonstrates that the hybrid composite presents an improved denture base material, based on its enhanced mechanical and tribological properties. The scope of this work was necessarily defined by several parameters: the specific nanoparticle concentrations investigated, a primary focus on in-vitro mechanical and tribological performance, and a laboratory-scale synthesis process; biological compatibility studies are designated for subsequent research. To build a complete material profile, future investigations will incorporate Fourier-transform infrared spectroscopy (FTIR), thermogravimetric analysis (TGA), and impact toughness testing. These analyses will critically elucidate the chemical interactions, thermal stability, and fracture resistance of the composite.

## Data Availability

The datasets used and analyzed during the current study are available from the corresponding author on reasonable request.
